# The relationship between objectification and the desire to undergo cosmetic surgery: the mediating role of intuitive eating and body image flexibility

**DOI:** 10.3389/fnut.2025.1537433

**Published:** 2025-06-13

**Authors:** Gianina Lăzărescu, Mona Vintilă

**Affiliations:** Faculty of Sociology and Psychology, West University of Timișoara, Timișoara, Romania

**Keywords:** cosmetic surgery, body objectification, intuitive eating, body flexibility, cosmetic surgery motivations

## Abstract

**Objectives:**

Sociocultural standards that emphasize idealized appearance and promote the objectification of women’s bodies have been consistently associated with negative body image outcomes and increased interest in cosmetic surgery (Fredrickson & Roberts; Calogero et al.). Constructs such as body image flexibility and intuitive eating have been discussed in the literature as psychological resources that promote well-being in body image–related contexts (Sandoz et al.; Tylka & Kroon Van Diest). However, their specific role in the relationship between objectification and cosmetic surgery interest remains underexplored. Recent evidence suggests that body image flexibility may function as a protective factor in this relationship (Huang et al.), while intuitive eating has been associated with greater psychological well-being and reduced body-related distress (Tylka and Wilcox), suggesting its potential relevance. Building on this background, the present study investigates the mediating roles of body image flexibility and intuitive eating in the relationship between body objectification and the desire to undergo cosmetic surgery for intrapersonal and social reasons, as well as the likelihood of pursuing such procedures in the future.

**Methods:**

The sample consisted of 555 Romanian women (M = 29.61 years, SD = 13.396), who completed validated scales measuring body objectification, body image flexibility, intuitive eating, and attitudes toward cosmetic surgery. Data were analyzed using parallel mediation models, controlling for age, educational status, ethnicity, relationship status, and body mass index.

**Findings:**

The findings indicated that body objectification was negatively associated with body image flexibility and intuitive eating. While intuitive eating did not mediate the relationship between body objectification and the desire for cosmetic surgery in any of the tested models, body image flexibility emerged as a partial mediator in the model related to social motivations and a full mediator in the model predicting future consideration of cosmetic procedures. These results are consistent with theoretical frameworks emphasizing self-perception and sociocultural context—such as self-verification and self-affirmation theories—that help explain how women’s behaviors are shaped by societal expectations and patriarchal cultural norms.

**Conclusion and recommendations:**

The study highlights the relevance of cultural context in understanding adaptive factors that may buffer the psychological impact of objectification. The results suggest that body image flexibility may function as a protective factor in reducing the desire for cosmetic surgery. While these findings may suggest potential directions for intervention, such as promoting positive body image and intuitive eating, we emphasize that further longitudinal research is needed before such psychoeducational programs can be designed or implemented. This study contributes to the growing body of literature by shedding light on culturally specific dynamics influencing cosmetic surgery motivations.

## Introduction

From time immemorial, society has played a significant role in transmitting culturally accepted ideals of beauty. The sociocultural perspective emphasizes the importance of physical appearance in Western cultures, with the predominant beauty ideal for women being thinness (1–3). The societal pressure to conform to culturally imposed body ideals has been consistently linked to various negative outcomes, including body dissatisfaction (4, 5), eating disorders (6, 7), low self-esteem (8, 9), the desire to undergo cosmetic surgery (10, 11), and self-objectification (12, 13). Objectification Theory (14) describes how social norms and representations can lead women to internalize an observer’s perspective of their own bodies, resulting in self-objectification. This process is associated with increased body surveillance, feelings of shame and guilt, and negative perceptions of body image (15–18, 112). According to this framework, women are often perceived more as objects than as individuals, and their bodies become instruments for the pleasure and evaluation of others (19, 20). Experiences of sexual objectification have been associated with body surveillance and body shame, which, in turn, are linked to positive attitudes toward cosmetic surgery (15, 21).

Although objectification is a widespread phenomenon, cultural context plays an important role in how body-related pressures are expressed and internalized. In Romania, the post-socialist legacy has contributed to the persistence of traditional gender norms, especially after 1989, when women were redefined primarily as caregivers and esthetic figures, and the pursuit of gender equality was deprioritized (22–24). The liberalization of the market economy after communism reinforced consumerist ideals, positioning physical appearance—particularly for women—as a key indicator of value and success (25, 26). These historical and cultural processes have shaped a context in which women are often evaluated based on their physical appearance. At the same time, Romanian public discourse continues to reflect patriarchal expectations and sexualized portrayals of women. Scholars have highlighted how patriarchal ideologies are sustained through norms and imagery that reinforce traditional roles and the objectification of women (27, 28). For example, Kaser (29) notes the frequent use of overtly sexualized representations of women in Romanian media, while Frunza et al. (30) document the use of hypersexualized imagery in advertising. These dynamics contribute to a socio-cultural environment in which women’s appearance is emphasized and objectifying messages are normalized.

One psychological variable that has received attention in relation to body image is body image flexibility, a construct grounded in Acceptance and Commitment Therapy (31). It refers to an individual’s capacity to experience negative body-related thoughts and emotions without attempting to suppress or change them (32). Individuals with high body image flexibility can respond to these experiences with openness and self-compassion rather than avoidance or criticism (33, 34). This construct has been studied in connection with body image difficulties (35) and disordered eating (36). A systematic review by Rogers et al. (37) identified associations between body image flexibility and lower levels of eating disorder symptoms, appearance concerns, and psychological distress, as well as higher levels of intuitive eating and self-compassion. It has also been examined as a mediator between negative emotional experiences and behavioral outcomes such as binge eating and BMI (38), and in relation to body appreciation among women with low BMI (39). Webb (40) found that body image flexibility partially explained the relationship between body dissatisfaction and body appreciation. In the context of objectification, Huang et al. (41) showed that body image inflexibility mediated the relationship between self-objectification and the intention to undergo cosmetic surgery. Individuals with lower body image flexibility may respond to body-related distress through maladaptive coping strategies, including cosmetic surgery, as a way to reduce unfavorable comparisons and internal discomfort (42).

A second construct of interest in this study is intuitive eating, which refers to eating based on internal physiological cues rather than external or emotional ones (43). The model includes four components: unconditional permission to eat, eating for physical rather than emotional reasons, reliance on internal hunger and fullness cues, and making food choices in line with the body’s needs (44, 45). Intuitive eating has been associated with lower engagement in unhealthy dietary behaviors and eating disorder symptoms (46–48), reduced pressure to maintain a thin ideal (49, 50), and more positive body image (51–54). Other findings point to associations with higher well-being and lower BMI (55, 56). Although intuitive eating has been associated with constructs relevant to objectification theory, including lower body surveillance, reduced internalization of appearance ideals, and greater body appreciation (57, 58), its mediating role in the link between objectification and cosmetic surgery interest remains unexplored. Additionally, intuitive eating has been found to be negatively associated with objectification experiences (59, 60), suggesting its potential relevance in understanding appearance-related investment behaviors.

**Figure 1:**
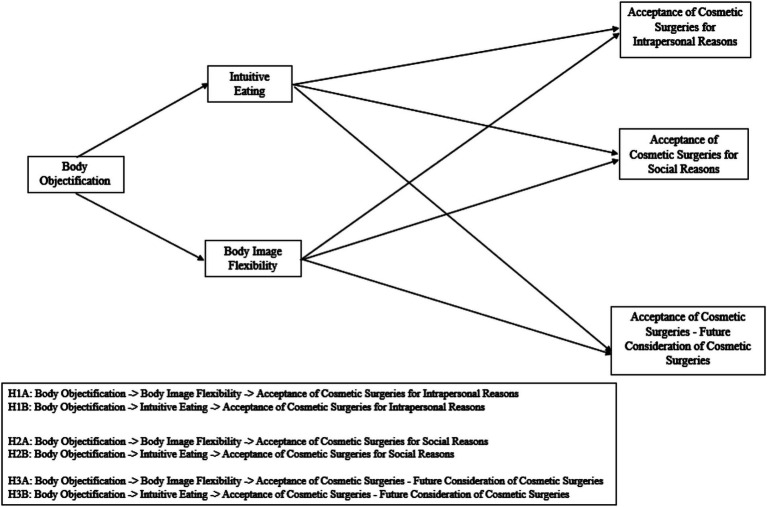
Parallel mediation models illustrating the hypothesized relationships between body objectification, intuitive eating, body image flexibility, and dimensions of cosmetic surgery acceptance (ACSS).

Considering the broader cultural context characterized by persistent patriarchal influences and the hypersexualization of women documented in Romania (29, 30), alongside the potential protective roles of body image flexibility and intuitive eating, the present study examines whether these constructs mediate the relationship between objectification and the desire to undergo cosmetic surgery. Building on the findings of Huang et al. (41), this study includes both body image flexibility and intuitive eating as parallel mediators. In addition, we examine three specific outcomes: intrapersonal reasons for cosmetic surgery, social reasons, and future consideration. To our knowledge, no prior study has investigated these mediators simultaneously within a Romanian sample. The proposed mediation models and hypotheses are presented in Figure 1.

*H*1*a*. Higher levels of body objectification are expected to be associated with lower levels of body image flexibility, which in turn may be related to a stronger intrapersonal desire to undergo cosmetic surgery.

*H*2*a*. Higher levels of body objectification are expected to be associated with lower levels of body image flexibility, which in turn may be related to a stronger social desire to undergo cosmetic surgery.

*H*3*a*. Higher levels of body objectification are expected to be associated with lower levels of body image flexibility, which in turn may be related to a greater likelihood of considering cosmetic surgery in the future.

*H*1*b*. Higher levels of body objectification are expected to be associated with lower levels of intuitive eating, which in turn may be related to a stronger intrapersonal desire to undergo cosmetic surgery.

*H*2*b*. Higher levels of body objectification are expected to be associated with lower levels of intuitive eating, which in turn may be related to a stronger social desire to undergo cosmetic surgery.

*H*3*b*. Higher levels of body objectification are expected to be associated with lower levels of intuitive eating, which in turn may be related to a greater likelihood of considering cosmetic surgery in the future.

## Materials and methods

### Participants

The sample consisted of 555 female participants aged between 18 and 79 years (M = 29.61; SD = 13.396). Of these, 69.91% identified as belonging to the ethnic majority, which in the Romanian context refers to ethnic Romanians. A total of 15.32% identified as belonging to an ethnic minority, and 14.77% did not give an answer to the ethnicity item. Participants were asked to indicate whether they belonged to an ethnic majority or an ethnic minority based on predefined response options. These options did not include specific categories for ethnic minority groups. This design choice aimed to maintain participant anonymity and reduce potential discomfort associated with disclosing sensitive identity-related information (61). Regarding education, 42.34% identified as students, 29.01% had completed undergraduate studies, 14.05% had completed secondary education, 13.33% had completed postgraduate studies, and 0.90% had completed primary education. In terms of marital status, 37.66% reported being in a non-marital relationship, 36.94% reported being single, 22.88% reported being married, and 0.72% reported being divorced or widowed.

### Measures

*Intuitive Eating* was measured using the Intuitive Eating Scale-2 (IES-2; (62)), in its version validated for the Romanian population (63). The scale consists of 23 items assessing the presence of this behavior in participants’ daily lives on a Likert scale ranging from 1 (strongly disagree) to 5 (strongly agree). The internal consistency of the scale in the current study was 0.91, 95% [CI = 0.90, 0.92].

*Body Image Flexibility*. The Body Image-Acceptance and Action Questionnaire (BI-AAQ; (32)) was used to measure this variable. The questionnaire consists of 12 items that assess various indicators related to body image, such as body image flexibility and the ability to experience and accept thoughts, beliefs, and feelings about one’s body. Items are rated on a Likert scale from 1 (never true) to 7 (always true), with the total score calculated by summing responses to all items. Items reflecting inflexibility were reverse-coded, so higher scores indicate greater body image flexibility. The scale demonstrated an internal consistency of 0.94, 95% [CI = 0.93, 0.95].

*Interest in Cosmetic Surgery* was assessed using the Acceptance of Cosmetic Surgery Scale (64), in its version validated for the Romanian population (65). The scale comprises 15 items measured on a Likert scale from 1 (strongly disagree) to 7 (strongly agree), investigating respondents’ attitudes toward cosmetic surgery as well as their self-reported likelihood of undergoing such procedures in the future. The three subscales corresponding to the factors identified in the psychometric validation on the Romanian population demonstrated the following internal consistencies in the current study: ACSS – Intrapersonal: 0.90, 95% [CI = 0.88, 0.91]; ACSS – Social: 0.86, 95% [CI = 0.83, 0.88]; ACSS – Consider: 0.93, 95% [CI = 0.91, 0.94].

*Body Objectification* was measured using the Objectified Body Consciousness Scale (OBCS; (66)), which consists of 24 items assessing women’s attitudes toward their bodies in relation to cultural beauty standards. The scale includes three subscales (Body Surveillance, Body Shame, and Control), with items rated on a Likert scale from 1 (strongly disagree) to 7 (strongly agree). Scores are calculated by summing the responses within each subscale and across the entire scale, with higher scores indicating higher levels of the measured constructs. The scale demonstrated an internal consistency of 0.79, 95% CI [0.76, 0.82].

Since no validated Romanian versions were available, the Objectified Body Consciousness Scale (OBCS) and the Body Image-Acceptance and Action Questionnaire (BI-AAQ) were translated using the 5-step adaptation procedure recommended by Beaton et al. (67). This included forward translation, synthesis, back-translation, expert committee review, and informal testing on a small group of Romanian women to ensure clarity and semantic accuracy. The final versions demonstrated good internal consistency (Cronbach’s *α* = 0.79 for the OBCS and α = 0.94 for the BI-AAQ).

### Procedure

Prior to conducting this research, ethical approval was obtained from the Ethics Committee of West University of Timișoara (approval code: 88120/23.11.2023). The study was conducted in accordance with the principles of the Declaration of Helsinki.

Participants were recruited using a non-probability snowball sampling method, which was appropriate given the exploratory nature of the study and the lack of access to a centralized sampling frame for Romanian adult women. The initial pool of participants was identified through the academic and professional networks of the authors, including university students, colleagues, and collaborators in the fields of psychology, health, and education. The survey was hosted on Google Forms and disseminated exclusively online.

No paid advertisements were used in the recruitment process. The survey link was shared organically via social media platforms, including public and private Facebook groups, discussion forums, and mailing lists. These included communities focused on topics such as body image, self-acceptance, and psychological well-being, as well as general-interest groups for women. Most participants were reached through networks centered in the Timișoara region, which likely resulted in a sample composed predominantly of individuals from urban areas. No data were collected regarding participants’ specific place of residence (urban vs. rural), as the recruitment process did not target or reach a geographically or residentially diverse population. This strategy nevertheless allowed for broad thematic dissemination of the study.

Potential participants were provided with detailed information about the study requirements and were assured of data confidentiality, anonymity, and the exclusive use of their responses for scientific research purposes. They were also informed of the estimated time needed to complete the survey (20–25 min) and reminded of their right to withdraw at any time if they experienced discomfort or distress.

Informed consent was obtained through a digital form prior to participation. Participants then completed the anonymous questionnaire, which included the validated scales described above. No financial compensation was offered. Before completing the scales, participants were asked to optionally provide their email addresses if they were willing to be contacted for future studies. Data collection took place between November and December 2023. Internet Protocol (IP) addresses were verified to ensure that no participant completed the survey multiple times.

## Results

### Preliminary analyses

Table 1 presents the central tendency indicators (mean and standard deviation) for the variables included in the study. Prior to conducting the mediation analyses, all assumptions were tested. Linearity was assessed through scatterplots with fitted regression lines between predictor, mediator, and outcome variables, confirming the presence of linear relationships.

**Table 1:** Cronbach’s alpha coefficient values and descriptive statistics for the analyzed variables.

Variable	Cronbach’s alpha	M	Minim and maximum possible variable values	SD	Skewness	Kurtosis
Body Objectification (OBCS)	0.79	97.33	24–168	17.61	0.108	0.351
Body Image Flexibility (BI-AAQ)	0.94	58.02	12–84	18.70	−0.513	−0.687
Intuitive Eating (IES-2)	0.91	83.44	23–115	15.99	−0.203	−0.516
Acceptance of Cosmetic Surgeries for Intrapersonal Reasons (ACSS – I)	0.90	21.33	5–35	7.97	−0.211	−0.666
Acceptance of Cosmetic Surgeries for Social Reasons (ACSS – S)	0.86	17.74	5–35	9.01	0.151	−1.184
Acceptance of Cosmetic Surgeries, future consideration of cosmetic surgeries (ACSS – C)	0.93	14.72	5–35	7.10	0.660	−0.198

M, Mean; SD, Standard Deviation; ACSS – I, Acceptance of Cosmetic Surgeries for Intrapersonal reasons; ACSS – S, Acceptance of Cosmetic Surgeries for Social reasons; ACSS – C, Acceptance of Cosmetic Surgeries, future consideration of cosmetic surgery.

Normality of distributions was evaluated by examining skewness and kurtosis values. As shown in Table 1, all variables exhibited values within commonly accepted thresholds—skewness within ±2 and kurtosis within ±7—based on guidelines drawn from applied psychometric literature (68, 69). Regardless of the guideline applied, our variables met the criteria for acceptable normality. Homoscedasticity was tested through the visual inspection of residual scatterplots, which showed no pattern indicating heteroscedasticity. These results support the robustness of the regression-based mediation approach used in this study. Additionally, we examined the potential for multicollinearity between the two mediators—intuitive eating and body image flexibility. The Pearson correlation coefficient between them was *r* = 0.60, *p* < 0.001, indicating a moderate relationship that does not exceed conventional thresholds for multicollinearity concerns (r > 0.70; (70, 71)). To further ensure the robustness of the model, Variance Inflation Factor (VIF = 1.587) and Tolerance (0.630) values were calculated in SPSS, both falling within acceptable limits (VIF < 5, Tolerance > 0.10; (72)). These results confirm that multicollinearity was not present and that the two mediators contributed distinct information to the analysis. Significant correlations were observed among all study variables (See Table 2).

**Table 2:** Intercorrelations among study variables.

Variable	OBCS	BI-AAQ	IES-2	ACSS – I	ACSS – S	ACSS – C
OBCS		−0.62**	−0.50**	0.22**	0.35**	0.28**
BI-AAQ			0.60**	−0.16**	−0.33**	−0.32**
IES-2				−0.09*	−0.25**	−0.21**
ACSS – I					0.75**	0.67**
ACSS – S						0.73**
ACSS – C						

**p < 0.01, *p < 0.05; OBCS, Body Objectification; BI-AAQ, Body Image Flexibility; IES-2, Intuitive Eating; ACSS – I, Acceptance of Cosmetic Surgeries for Intrapersonal reasons; ACSS – S, Acceptance of Cosmetic Surgeries for Social reasons; ACSS – C, Acceptance of Cosmetic Surgeries, future consideration of cosmetic surgery.

A parallel mediation model was constructed, in which each of the three factors of the ACSS scale (Intrapersonal, Social, and Consider) was treated as the dependent variable in turn. Scores on the body image flexibility and intuitive eating scales were considered mediators, while body objectification was treated as the independent variable. Model 4 of the PROCESS extension, version 4.2 for SPSS (73), was used for the analysis.

To account for the influence of extraneous variables, age, educational status, ethnicity, relationship status, and body mass index were controlled as covariates in each analyzed model. Following Hayes’s (113) recommendation, the bootstrap sample size was set at 10,000, with a 95% confidence interval in PROCESS to ensure the stability of each proposed mediation model. A statistically significant effect was noted when 0 was not included within the confidence intervals (74). In this study, effect sizes were evaluated through standardized regression coefficients (*β*) and indirect effects with 95% bias-corrected bootstrap confidence intervals, consistent with the recommendations for mediation analysis.

### Testing parallel mediation models

The first parallel mediation model was constructed with body objectification as the independent variable, intuitive eating and body image flexibility as mediators, and the desire to undergo cosmetic surgery for intrapersonal reasons as the dependent variable. Age, educational status, ethnicity, relationship status, and body mass index were included as covariates and controlled for in the analysis. Body objectification was negatively associated with body image flexibility (*β* = −0.55, *p* < 0.01). However, no significant association was found between body image flexibility and the desire to undergo cosmetic surgery for intrapersonal reasons (*β* = −0.08, *p* = 0.169). Mediation analysis indicated that body image flexibility did not mediate the relationship between body objectification and the desire to undergo cosmetic surgery for intrapersonal reasons [indirect effect 1 = 0.04, CIs = (−0.02, 0.01)]. Similarly, body objectification was negatively associated with intuitive eating (*β* = −0.44, *p* < 0.001), but no significant association was found between intuitive eating and the desire to undergo cosmetic surgery for intrapersonal reasons (*β* = 0.03, *p* = 0.578). Thus, intuitive eating also did not mediate the relationship between body objectification and the desire to undergo cosmetic surgery for intrapersonal reasons [indirect effect 2 = −0.01, CIs = (−0.06, 0.03)]. A positive direct relationship was observed between body objectification and the desire to undergo cosmetic surgery for intrapersonal reasons (*β* = 0.06, *p* < 0.05), with a total effect of *β* = 0.08, *p* < 0.01.

The second parallel mediation model was constructed with body objectification as the independent variable, intuitive eating and body image flexibility as mediators, and the desire to undergo cosmetic surgery for social reasons as the dependent variable. Age, educational status, ethnicity, relationship status, and body mass index were again included as covariates. Body objectification was negatively associated with body image flexibility (*β* = −0.55, *p* < 0.001), and body image flexibility was negatively associated with the desire to undergo cosmetic surgery for social reasons (*β* = −0.16, *p* < 0.01). Mediation analysis revealed that body image flexibility mediated the relationship between body objectification and the desire to undergo cosmetic surgery for social reasons [indirect effect 1 = 0.09, CIs = (0.02, 0.16)]. Meanwhile, body objectification was negatively associated with intuitive eating (*β* = −0.44, *p* < 0.001), but no significant association was found between intuitive eating and the desire to undergo cosmetic surgery for social reasons (*β* = −0.04, *p* = 0.390). Thus, intuitive eating did not mediate the relationship between body objectification and the desire to undergo cosmetic surgery for social reasons [indirect effect 2 = 0.02, CIs = (−0.03, 0.07)]. Given the positive association between body objectification and the desire to undergo cosmetic surgery for social reasons (*β* = 0.09, *p* < 0.01), it can be concluded that body image flexibility partially mediates this relationship. The total effect was *β* = 0.15, *p* < 0.001, and the total indirect effect was also significant [indirect total = 0.11, CIs = (0.05, 0.18)].

The third parallel mediation model was constructed with body objectification as the independent variable, intuitive eating and body image flexibility as mediators, and the likelihood of undergoing cosmetic surgery in the future as the dependent variable. Age, educational status, ethnicity, relationship status, and body mass index were included as covariates and controlled for in the analysis.

Body objectification was negatively associated with body image flexibility (*β* = −0.55, *p* < 0.001), and body image flexibility was negatively associated with the likelihood of undergoing cosmetic surgery in the future (*β* = −0.28, *p* < 0.001). Mediation analysis indicated that body image flexibility mediated the relationship between body objectification and the likelihood of undergoing cosmetic surgery in the future [indirect effect 1 = 0.15, CIs = (0.09, 0.22)].

Simultaneously, body objectification was negatively associated with intuitive eating (*β* = −0.44, *p* < 0.001), but no significant association was found between intuitive eating and the likelihood of undergoing cosmetic surgery in the future (*β* = −0.01, *p* = 0.728). Thus, intuitive eating did not mediate the relationship between body objectification and the likelihood of undergoing cosmetic surgery in the future [indirect effect 2 = 0.001, CIs = (−0.03, 0.05)].

Since no direct associations were found between body objectification and the likelihood of undergoing cosmetic surgery in the future (*β* = 0.04, *p* > 0.05), it can be concluded that body image flexibility fully mediates the relationship between the two variables. The total effect was *β* = 0.10, *p* < 0.001, and the total indirect effect was also significant [indirect total = 0.16, CIs = (0.09, 0.23)].

## Discussion

The objective of this study was to examine whether body image flexibility and intuitive eating mediate the association between body objectification and the desire to undergo cosmetic surgery for intrapersonal and social reasons, as well as the likelihood of pursuing such interventions in the future, within an exclusively female population in Romania.

Across all tested models, body objectification was negatively associated with intuitive eating. Intuitive eating showed negative, yet non-significant, associations with the desire to undergo cosmetic surgery for social reasons and future intentions, and a positive, non-significant association with the intrapersonal motive. Consequently, intuitive eating did not emerge as a significant mediator in any of the proposed models.

This outcome may be understood in light of previous findings, which primarily link intuitive eating to eating behaviors and disorders (46–48). Individuals who engage in intuitive eating rely on physiological hunger and satiety cues to guide their food-related decisions (75). Moreover, intuitive eating is a relatively new concept, positioned as a psychologically adaptive strategy when compared to emotional or restrictive eating patterns (76), which are reportedly more prevalent among the Romanian population (77, 78).

Given the inconsistent levels of nutritional education in Romania (63, 79), it is plausible that participants were unfamiliar with this approach or less inclined to associate it with body-related investment strategies such as cosmetic surgery. It is also possible that intuitive eating, when employed, is more often linked to non-invasive appearance regulation behaviors such as healthy eating (80), gym participation (81), or maintaining a toned physique (82). Although intuitive eating was not found to be a significant mediator in the current study, this finding does not necessarily imply that the construct is irrelevant to appearance-related investment behaviors. While the mean score on the intuitive eating scale was above the minimum, suggesting some familiarity or openness to the construct, this does not guarantee consistent behavioral application or deep internalization of intuitive eating principles. As Avalos and Tylka (57) noted, intuitive eating is a multifaceted construct that requires both body trust and attitudinal alignment—not merely occasional reliance on hunger cues. In the Romanian cultural context, where appearance-focused norms and diet culture remain prevalent (63, 79), it is plausible that intuitive eating is not as deeply rooted or practiced as in populations where it has been more widely promoted or studied. Consequently, even moderate levels of intuitive eating may not be sufficient to act as a protective factor against objectification-related cosmetic surgery motivations in this sample (57). Future research using qualitative methods or subscale-level analysis could help clarify how intuitive eating is expressed and internalized in such cultural contexts.

Regarding body image flexibility, this construct was consistently and negatively associated with body objectification in all three models. Significant negative associations were also found between body image flexibility and the desire to undergo cosmetic surgery for social reasons and future intentions. The association with intrapersonal desire was negative but not statistically significant. As such, body image flexibility significantly mediated two of the three relationships tested.

These findings are consistent with prior research highlighting body image flexibility as a protective psychological resource. One possible explanation for these results lies in the social interaction model of objectification, which posits that individuals weigh the costs and benefits of objectifying interactions within patriarchal structures that grant men greater power and reduce women to bodily attributes (83). While men are known to objectify women through suggestive comments and gazes (84), women also engage in self-objectification through strategies such as provocative dress (85) or curated online appearances (86).

In this context, Romania remains shaped by persistent patriarchal norms and post-communist gender expectations (87). Following Romania’s accession to the European Union and neoliberal transitions, women have increasingly been positioned as hyper-feminine, sexualized subjects, especially in the media (28, 88, 89). The present findings align with these cultural patterns, suggesting that cosmetic surgery motivations may be rooted in sociocultural dynamics that reinforce appearance-based worth.

Theoretical frameworks such as self-verification (90), symbolic self-completion (91), and self-affirmation theory (92) further explain how individuals pursue identity-consistent behaviors. When objectification is normalized and perceived as socially rewarding, women may engage in body-altering practices as a means of reinforcing their self-image and gaining social validation (93).

These dynamics may help explain why lower levels of body image flexibility were associated with cosmetic surgery motivations, particularly for social reasons and future intentions. Recent studies have emphasized how social media environments amplify these pressures by promoting narrow beauty ideals and appearance-based comparison. Zaharia and Gonța (94) demonstrate that exposure to idealized body representations on social platforms contributes to internalization of unrealistic esthetic standards, body dissatisfaction, and compensatory behaviors aimed at reducing the gap between actual and ideal appearance. Similarly, Arab et al. (95) found that viewing cosmetic surgery-related content online and comparing oneself to influencers significantly increases the likelihood of considering cosmetic procedures, often driven by perceived social expectations rather than intrinsic desire. Franchina and Lo Coco (96) further argue that even positive social media feedback may reinforce body monitoring and concern, as individuals internalize the idea that their appearance is continuously evaluated. Taken together, these findings suggest that the pursuit of cosmetic enhancement is not merely esthetic, but reflects a deeper social logic of validation, symbolic affirmation, and identity maintenance. In contrast, the intrapersonal pathway was not statistically significant, potentially because such motivations relate more to self-worth and internal validation, which may not be fully captured by body image flexibility alone (97).

The desire to enhance physical appearance may also be fueled by broader interpersonal concerns, such as partner retention strategies (98–100) or intrasexual competition (101, 102). Previous research indicates that women may be more likely to invest in appearance-enhancing behaviors when competing for male attention (103).

Finally, our results complement those of Huang et al. (41), who found that body image inflexibility mediates the relationship between self-objectification and cosmetic surgery interest. Individuals with low flexibility may rely on maladaptive coping strategies to manage body-related distress and unfavorable comparisons (42), including cosmetic procedures aimed at restoring perceived body control and social value.

## Limitations and future research

The primary limitation of this study lies in its cross-sectional design, which does not allow for causal inferences. Longitudinal or experimental intervention studies are needed to examine how these variables change over time and to explore the potential of intuitive eating and body image flexibility as protective and adaptive factors in the relationship between objectification and the desire to undergo cosmetic surgery.

Another limitation concerns the composition of the sample. Most participants were recruited from the Timișoara region, which may reduce the geographical and cultural variability within the sample. Additionally, information regarding the participants’ residential environment (urban vs. rural) was not collected, although the recruitment methods likely reached predominantly urban individuals. These factors limit the generalizability of the findings to women from other regions of Romania or from rural settings, where sociocultural attitudes toward body image and cosmetic surgery may differ.

An additional limitation concerns the recruitment strategy. Participants were recruited online, primarily through Facebook groups oriented toward themes such as body image, self-acceptance, psychological wellbeing, or general women’s interests. Although the study invitation was framed broadly and did not explicitly mention cosmetic surgery or related constructs, it is possible that individuals already invested in appearance-related issues were more likely to participate. This self-selection may limit the generalizability of the findings to the broader female population, particularly to women who are less engaged with appearance or body image topics. Future research should consider broader and more diverse recruitment strategies to minimize potential sampling biases.

Furthermore, although the study included a wide age range of participants (18–79 years), the majority were younger women, with a mean age of 29.61 years. Age was statistically controlled for in all analyses to minimize potential confounding; however, objectification experiences and attitudes toward cosmetic surgery may vary across developmental stages. Therefore, future research could benefit from stratified analyses or targeted recruitment to explore age-specific patterns. Such investigations may help clarify whether the psychological mechanisms identified in this study operate differently across early, middle, and later adulthood.

Additionally, a limitation of the current study is the lack of detailed information regarding the specific composition of the ethnic minority group(s). Although ethnicity was controlled for in the analyses, the use of general ethnic categories (“majority” vs. “minority”) limits the possibility of exploring within-group differences among minority participants. Future research may benefit from including more granular ethnic identification, provided confidentiality can be ensured.

Moreover, the present study did not examine the specific motivations underlying participants’ interest in cosmetic surgery beyond the three general dimensions assessed (intrapersonal, social, and future intention). It is possible that intuitive eating may be more or less relevant depending on the reason for pursuing surgery—for example, motivations driven by body dissatisfaction may relate differently to eating behaviors than those rooted in social approval or appearance enhancement. Additionally, the study did not assess which specific body areas participants were interested in modifying through cosmetic surgery (e.g., face, breasts, and body fat). This distinction may be important, as dissatisfaction with weight-related areas could be more closely linked to eating behaviors and intuitive eating than dissatisfaction with features less influenced by weight. Future research should incorporate assessments of the targeted body areas to better understand how different types of appearance concerns may interact with psychological factors such as intuitive eating.

Another methodological consideration concerns the inclusion of body mass index (BMI) as a covariate in all mediation models. Although BMI was not a primary variable of interest, it has been associated in prior research with both objectification experiences and body image-related outcomes, including appearance investment and cosmetic surgery intentions. Therefore, we included it to statistically adjust for its potential confounding influence on the relationships under investigation. Similar analytic choices have been reported in previous studies examining body image and esthetic attitudes (104–106). However, we acknowledge the ongoing debate in the literature regarding the role of BMI as a potential mediator or collider variable in appearance-related research. Future studies might consider modeling BMI differently or comparing models with and without BMI as a covariate to better understand its function within these psychological processes. Sensitivity analyses were also conducted excluding BMI as a covariate. These analyses indicated that the exclusion of BMI did not substantively alter the direction, significance, or interpretation of the direct and indirect effects observed in the study.

Finally, all research instruments were self-report scales, which may introduce response bias due to the inability to control factors specific to the respondents (e.g., their level of motivation to respond, providing answers inconsistent with reality to appear socially desirable).

In future research, it would also be valuable to explore other psychological factors that may mediate or moderate the relationship between self-objectification and the desire to undergo cosmetic surgery. Variables such as self-esteem (107), body dysmorphic disorder symptoms (108), and perfectionism (109) have been associated with body image disturbances and esthetic investment. For instance, Mancin et al. (108) found that individuals with higher levels of body dysmorphic disorder symptoms were more likely to engage in photo-related behaviors, such as frequent photo editing or sharing, which reflect heightened appearance concerns and may be linked to motivations for cosmetic procedures. Incorporating such constructs into future models could provide a more comprehensive understanding of the psychological mechanisms underlying body-related distress and the interest in cosmetic surgery.

Moreover, although this study tested three separate mediation models using different dependent variables, we did not apply formal correction procedures for Type I error (e.g., Bonferroni). Each model was theory-driven and addressed a distinct dimension of cosmetic surgery motivations. As Hayes (73) notes, applying overly strict correction methods in theoretically grounded models can unnecessarily inflate the risk of Type II errors. Additionally, the use of 10,000 bootstrap resamples offers a robust method for estimating indirect effects, reducing the likelihood of spurious findings. Future research may benefit from replication using preregistered designs or confirmatory models in independent samples.

## Theoretical and practical implications

This study underscores the importance of integrating cultural context when examining the psychological mechanisms linking body objectification to cosmetic surgery motivations. Rather than attempting to validate an existing theoretical model, our aim was to explore the applicability of concepts such as body image flexibility and intuitive eating within a Romanian sample—a population underrepresented in this area of research. Given Romania’s distinct sociocultural backdrop, shaped by both patriarchal values and post-communist gender dynamics, we argue that theoretical frameworks developed in Western contexts may not generalize seamlessly across cultures. As such, testing whether these mechanisms operate similarly in non-Western or transitional societies is a necessary step toward building culturally sensitive models of appearance-related investment. Given Romania’s specific sociocultural context, including its evolving post-communist identity and the influence of traditional gender expectations, the use of culturally adapted and semantically coherent measures is essential (110). This ensures that psychological constructs retain their theoretical integrity across languages and contexts. Our findings offer preliminary insights into how these psychological constructs manifest in a Romanian context and highlight the need for further cross-cultural investigations.

At the same time, psychotherapists, counselors, and school psychologists can develop prevention, psychoeducation, and intervention programs to emphasize the negative aspects of objectification in societies where such behavior is normalized. In this context, effective communication strategies are essential to ensure that messages related to body functionality, self-acceptance, and intuitive eating are accessible and impactful (111). Children and adolescents can thus learn from an early age to appreciate their bodies for their functionality rather than what they can offer to others. Educating individuals about intuitive eating can serve as a valuable starting point for fostering a positive relationship with their bodies. However, it is important to note that, in the present study, intuitive eating did not emerge as a significant mediator, and thus these recommendations should be considered preliminary and subject to further empirical validation in future research.

## Conclusion

In a society that normalizes objectification and places significant emphasis on physical appearance, it is crucial to investigate factors that can counteract the harmful effects of these behaviors. Promoting a positive relationship with one’s body by appreciating it for its functionality and fostering trust in the signals it provides regarding its needs can serve as precursors to enhanced psychological and physical well-being.

## Data Availability

The original contributions presented in the study are included in the article/Supplementary material, further inquiries can be directed to the corresponding author.
