# Supplementary material for: The relationship between objectification and the desire to undergo cosmetic surgery: the mediating role of intuitive eating and body image flexibility
Source: Front Nutr. 2025 Jun 13;12:1537433. doi: 10.3389/fnut.2025.1537433 (PMC12202425; doi:10.3389/fnut.2025.1537433)
Supplement: Supplementary file 1 [file Table_1.docx]

# Annex no. 1 – Variables from the tested model

| Variable / Construct | Item from Questionnaire | Scale | Adapted from |
| --- | --- | --- | --- |
| Intuitive Eating (IES-2) | I try to avoid certain foods high in fat, carbohydrates, or calories. | 1–5 (1 = Strongly disagree; 5 = Strongly agree) | Tylka & Kroon Van Diest (2013); Vintilă et al. (2020) |
|  | I find myself eating when I’m feeling emotional (e.g., anxious, sad, or bored), even when I’m not physically hungry. |  |  |
|  | If I am craving a certain food, I allow myself to have it. |  |  |
|  | I get mad at myself for eating something unhealthy. |  |  |
|  | I trust my body to tell me when to eat. |  |  |
|  | I have forbidden foods that I don’t allow myself to eat. |  |  |
|  | I find myself eating when I am lonely, even when I’m not physically hungry. |  |  |
|  | I trust my body to tell me what to eat. |  |  |
|  | I don’t keep certain foods in my house/apartment because I think that I may lose control and eat them. |  |  |
|  | I can tell when I’m slightly hungry. |  |  |
|  | I find myself eating when I’m stressed out, even when I’m not physically hungry. |  |  |
|  | I allow myself to eat what I desire, even if I’m not sure it’s a healthy choice. |  |  |
|  | I stop eating when I feel full. |  |  |
|  | I don’t allow myself to eat what food I most desire. |  |  |
|  | I find myself eating when I’m bored, even when I’m not physically hungry. |  |  |
|  | I rely on my hunger signals to tell me when to eat. |  |  |
|  | I feel guilty if I eat a food that I consider unhealthy. |  |  |
|  | I pay attention to how foods affect my body. |  |  |
|  | I try to avoid foods that I used to enjoy because they are unhealthy. |  |  |
|  | I find myself eating when I’m sad, even when I’m not physically hungry. |  |  |
|  | I listen to my body to tell me what to eat. |  |  |
|  | I feel that eating healthy foods is the most important aspect of my diet. |  |  |
|  | I honor my hunger signals. |  |  |
| Interest in Cosmetic Surgery (ACSS) | Cosmetic surgery can be a big benefit to people’s self-image. | 1–7 (1 = Strongly disagree; 7 = Strongly agree) | Henderson-King & Henderson-King (2005); Lăzărescu et al. (2023) |
|  | It makes sense to have small cosmetic procedures rather than live with the flaw. |  |  |
|  | Cosmetic surgery is a good thing because it can help people feel better about themselves. |  |  |
|  | Cosmetic surgery is a good investment if you want to maintain your youth. |  |  |
|  | Cosmetic surgery is a good option when one wants to make a change in their appearance. |  |  |
|  | If it would benefit my career, I would think about having cosmetic surgery. |  |  |
|  | I would never consider having any kind of cosmetic surgery. |  |  |
|  | If I knew there were no negative side effects or pain, I would like to try cosmetic surgery. |  |  |
|  | I have sometimes thought about having cosmetic surgery. |  |  |
|  | I would consider having cosmetic surgery in the future. |  |  |
|  | I would like to undergo cosmetic surgery if it could make me feel more attractive. |  |  |
|  | I would not be embarrassed if people knew I had undergone cosmetic surgery. |  |  |
|  | I would seriously consider having cosmetic surgery if my physical appearance bothered me. |  |  |
|  | If someone gave me cosmetic surgery as a gift, I would consider having it. |  |  |
|  | I would never consider having plastic surgery, even if it were free. |  |  |
| Body Objectification (OBCS) | I rarely think about how I look. | 1–7 (1 = Strongly disagree; 7 = Strongly agree) | McKinley & Hyde (1996) |
|  | I think it is more important that my clothes are comfortable than whether they look good on me. |  |  |
|  | I think more about how my body feels than how my body looks. |  |  |
|  | I rarely compare how I look with how other people look. |  |  |
|  | During the day, I think about how I look many times. |  |  |
|  | I often worry about whether the clothes I am wearing make me look good. |  |  |
|  | I rarely worry about how I look to other people. |  |  |
|  | I am more concerned with what my body can do than how it looks. |  |  |
|  | When I can’t control my weight, I feel like something must be wrong with me. |  |  |
|  | I feel ashamed of myself when I haven’t made the effort to look my best. |  |  |
|  | I feel like I must be a bad person when I don’t look as good as I could. |  |  |
|  | I would be ashamed for people to know what I really weigh. |  |  |
|  | I never worry that something is wrong with me when I am not exercising as much as I should. |  |  |
|  | When I’m not exercising enough, I question whether I am a good enough person. |  |  |
|  | Even when I can’t control my weight, I think I’m an okay person. |  |  |
|  | When I’m not the size I think I should be, I feel ashamed. |  |  |
|  | I think a person is pretty much stuck with the looks they are born with. |  |  |
|  | A large part of being in shape is having that kind of body in the first place. |  |  |
|  | I think a person can look pretty much how they want to if they are willing to work at it. |  |  |
|  | I really don’t think I have much control over how my body looks. |  |  |
|  | I think a person’s weight is mostly determined by the genes they are born with. |  |  |
|  | It doesn’t matter how hard I try to change my weight, it’s probably always going to be about the same. |  |  |
|  | I can weigh what I’m supposed to when I try hard enough. |  |  |
|  | The shape you are in depends mostly on your genes. |  |  |
| Body Image Flexibility (BI-AAQ) | I worry about my weight. | 1–7 (1 = Never true; 7 = Always true) | Sandoz et al. (2013) |
|  | My thoughts and feelings about my body prevent me from doing what I would otherwise do. |  |  |
|  | I become self-conscious about my body in public. |  |  |
|  | Worrying about my weight makes it difficult for me to live a life that I value. |  |  |
|  | I will not go out and do things that I enjoy if I feel bad about the way that I look. |  |  |
|  | My appearance is the most important part of who I am. |  |  |
|  | I stop doing things that are important to me whenever I feel bad about my body. |  |  |
|  | I worry about what other people think about my body. |  |  |
|  | My self-worth is independent of my body shape or weight. |  |  |
|  | I feel pressure to be thin from people in my life. |  |  |
|  | Feeling fat causes problems in my life. |  |  |
|  | If I feel unattractive, I avoid situations where others might see me. |  |  |
